# The validity of electronic health data for measuring smoking status: a systematic review and meta-analysis

**DOI:** 10.1186/s12911-024-02416-3

**Published:** 2024-02-02

**Authors:** Md Ashiqul Haque, Muditha Lakmali Bodawatte Gedara, Nathan Nickel, Maxime Turgeon, Lisa M. Lix

**Affiliations:** 1 Department of Community Health Sciences, University of Manitoba, Winnipeg, MB Canada; 2 Department of Statistics, University of Manitoba, Winnipeg, MB Canada

**Keywords:** Algorithms, Electronic health records, Review, Routinely collected health data, Validation study

## Abstract

**Background:**

Smoking is a risk factor for many chronic diseases. Multiple smoking status ascertainment algorithms have been developed for population-based electronic health databases such as administrative databases and electronic medical records (EMRs). Evidence syntheses of algorithm validation studies have often focused on chronic diseases rather than risk factors. We conducted a systematic review and meta-analysis of smoking status ascertainment algorithms to describe the characteristics and validity of these algorithms.

**Methods:**

The Preferred Reporting Items for Systematic Reviews and Meta-Analyses guidelines were followed. We searched articles published from 1990 to 2022 in EMBASE, MEDLINE, Scopus, and Web of Science with key terms such as validity, administrative data, electronic health records, smoking, and tobacco use. The extracted information, including article characteristics, algorithm characteristics, and validity measures, was descriptively analyzed. Sources of heterogeneity in validity measures were estimated using a meta-regression model. Risk of bias (ROB) in the reviewed articles was assessed using the Quality Assessment of Diagnostic Accuracy Studies-2 tool.

**Results:**

The initial search yielded 2086 articles; 57 were selected for review and 116 algorithms were identified. Almost three-quarters (71.6%) of algorithms were based on EMR data. The algorithms were primarily constructed using diagnosis codes for smoking-related conditions, although prescription medication codes for smoking treatments were also adopted. About half of the algorithms were developed using machine-learning models. The pooled estimates of positive predictive value, sensitivity, and specificity were 0.843, 0.672, and 0.918 respectively. Algorithm sensitivity and specificity were highly variable and ranged from 3 to 100% and 36 to 100%, respectively. Model-based algorithms had significantly greater sensitivity (*p* = 0.006) than rule-based algorithms. Algorithms for EMR data had higher sensitivity than algorithms for administrative data (*p* = 0.001). The ROB was low in most of the articles (76.3%) that underwent the assessment.

**Conclusions:**

Multiple algorithms using different data sources and methods have been proposed to ascertain smoking status in electronic health data. Many algorithms had low sensitivity and positive predictive value, but the data source influenced their validity. Algorithms based on machine-learning models for multiple linked data sources have improved validity.

**Supplementary Information:**

The online version contains supplementary material available at 10.1186/s12911-024-02416-3.

## Background

Electronic health databases, including electronic medical records (EMRs) and administrative data, contain routinely-collected information that is widely used for health research [1, 2] even though they were not originally intended for this purpose. EMRs typically include a diverse amount of information about the patient, including medical history, family history, immunization status, laboratory test results, and radiology images [3]. Administrative data also include large amounts of information, including insurance enrollment dates, inpatient and outpatient contacts, and vital statistics [4]. Data quality is an important consideration when using electronic health databases for research, given that the data are used for secondary purposes.

Smoking is responsible for more than 8 million deaths worldwide each year [5] and is the leading cause of preventable diseases and premature deaths [6]. Smoking is a significant risk factor for cancers, cardiovascular diseases, and respiratory diseases. Valid measurement of smoking status contributes to accurate estimates from risk prediction models and other outcome studies about these diseases [7]. Valid smoking status measures can also aid in accurately estimating disease trends at the population level.

Population-based surveys are typically used to capture information about smoking status. However, they are expensive to conduct and are not always conducted on a routine basis [8]. Routinely collected administrative data often contain indirect information about smoking status, such as diagnosis codes for related diseases (e.g., chronic bronchitis) and substance use disorders. EMRs capture information on smoking status through free-text information about one’s health history as well as diagnosis codes. Therefore, many studies have investigated the validity of electronic health databases, including administrative databases and EMRs, for capturing information on smoking status. For example, a study based on Medicare claims data reported smoking status ascertainment algorithms with limited sensitivity but very high specificity [9]. In a different study, sensitivity estimates of algorithms for EMRs within an integrated healthcare system varied widely by years of data used [10]. However, the positive predictive value (PPV) consistently remained high.

To date, there have been few, if any studies that have systematically examined validation studies about smoking status in electronic health databases. Summary information about smoking status ascertainment algorithms might be used to develop recommendations about the optimal algorithm(s) to use and opportunities for further research. The latter is particularly timely, given increasing interest in novel approaches to mine new information from electronic health databases using machine-learning methods [11–13].

Given this background, the purpose of our study was to synthesize information about smoking status algorithms developed for electronic health databases. The objectives were to describe smoking status algorithm characteristics, methods to construct the algorithms, and estimates of their validity. A systematic review methodology was used to provide a comprehensive summary of the algorithms to ascertain smoking status [14]. Meta-analysis [15] of algorithm validity measure estimates was conducted to assess the potential sources of heterogeneity in them.

## Methods

We used the Preferred Reporting Items for Systematic Reviews and Meta-Analyses (PRISMA) guideline for this review [16] (see Additional file 4). This guideline is widely recognised for ensuring rigorous and consistent reporting in systematic reviews and meta-analyses [17].

### Data sources and search strategy

EMBASE, MEDLINE, Scopus, and Web of Science were searched from 1990 to November 22, 2022. The target sources were English-language peer-reviewed journal articles. We excluded review articles. The search strategy was developed by the research team in consultation with an experienced university librarian. The search strategy was based on three concepts, each built with specific sets of keywords. The concepts are validity measures (valid*, quality, accuracy, sensitivity, specificity), electronic health data (electronic medical record*, electronic health record*, administrative health data, administrative data, health care data, administrative billing record*, administrative claims data, claims data, hospital data, hospital discharge data, medicare data, medicaid data), and smoking status (smok*, tobacco). The * at the end of the words valid, record, and smok indicates the use of truncation to capture variant endings. The keywords within each set were connected with OR and the concepts were connected with AND. Article titles, abstracts, and keywords were reviewed to identify potentially relevant articles. The detailed search strategy implemented for each database is available in Additional file 1.

### Study selection

The titles, abstracts, and keywords of the selected articles were uploaded to Rayyan [18] for deduplication and screening for inclusion. An article was included if it reported the results of a validation study for one or more smoking status ascertainment algorithms developed for EMRs or administrative health data (e.g., hospital records, physician claims, prescription drug records). There were no restrictions on geography of the data or population characteristics (e.g., age, sex). To ensure an acceptable level (> 80%) [19] of agreement between the two reviewers who conducted the screening, two rounds of abstract and title screening training were undertaken; each training session was conducted on a random sample of 10% of the identified articles. Both reviewers independently screened all the articles. Inter-reviewer agreement was assessed with Cohen’s kappa [20]. Disagreements on study selection for full-text review were resolved by consensus. Articles were retained when there was uncertainty regarding the eligibility to be included based on title, abstract, and keywords alone. The final decision about the inclusion of an article in this study was made with full text review of the articles identified based on titles, abstracts, and keywords screening. The reference lists of the selected articles were searched for additional articles.

### Data extraction

Two reviewers independently extracted data from two randomly selected articles in a training session to maintain high inter-reviewer reliability [21]. Disagreements on data extraction were resolved by consensus and discussion between the reviewers. The remainder of the articles were equally distributed to the two reviewers for data extraction. We extracted information from the selected studies about article characteristics, algorithm characteristics, and algorithm validation estimates.

Article characteristics included year of publication, geographical data source, whether data from multiple jurisdictions were used, and journal discipline. The latter was determined based on subject terms from the United States National Library of Medicine catalog in PubMed.

Algorithm characteristics included International Statistical Classification of Diseases and Related Health Problems (ICD) codes, procedure or intervention codes, data source, data structure, and the use of a predictive model to develop the algorithm. The algorithm data source was categorized as EMR or administrative data. Data structure was classified as structured (e.g., diagnosis codes), unstructured text (e.g., clinical notes), or both structured and unstructured. Algorithms were classified as model-based or deterministic/rule-based on the basis of the method of construction [22]. Model-based algorithms implemented predictive statistical and/or machine-learning models (e.g., support vector machine). Rule-based approaches relied on measuring the type and frequency of diagnosis/billing codes in the records of an individual.

Information about the validation data source and validity measures (e.g., sensitivity, specificity) was also extracted from the articles. For model-based algorithms, the validity measure estimates for test data were extracted. The validation source was classified as self-reported data (e.g., survey), chart review data (e.g., patient charts reviewed to extract smoking status based on assessments by clinical or domain-knowledge experts), and clinical data (e.g., blood test results). The reported validity measures and their respective estimates and 95% confidence intervals (CI) were recorded. If estimates were reported for more than one sub-group or category (e.g., by demographic characteristics, by years of data), only the overall value of the validity measure was extracted. Finally, we assessed whether the Standards for Reporting Diagnostic Accuracy (STARD) criterion [23] about the number of measures recommended for reporting were fulfilled.

### Statistical analysis

We analyzed the extracted data at both the study level and algorithm level. At the study level, data about the articles were descriptively analyzed using frequencies and percentages. At the algorithm level, we conducted descriptive analyses overall, and then stratified by algorithm characteristics. The distributions of algorithm validity measures were visually summarized using boxplots; the median and interquartile range (IQR) were used to describe the data.

**Fig. 1:**
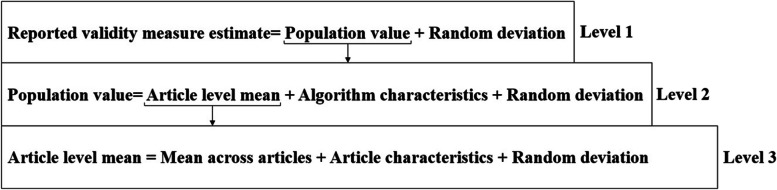
Three-level meta-regression model

Sources of heterogeneity in algorithm validity estimates, including algorithm characteristics and article characteristics, were examined using a three-level meta-regression model [24]. The first level accounts for sampling error, the second level examines algorithm characteristics, and the third level considers article characteristics. The structure of this model is depicted in Fig. 1. The random deviations at each level were assumed to follow a normal distribution with zero mean and constant variance.

Standard errors for the reported estimates were calculated from CIs or number of positive/negative cases in the validation data [25]. Likelihood ratio (LR) tests were conducted to compare three-level models against their two-level counterparts [26]. Null models were fitted to calculate pooled estimate and variance in the reported validity measures. To further investigate the sources of heterogeneity in these estimates, predictor variables were included in the model. At level 2, data source (1 = EMRs, 0 = Administrative) and the use of a predictive model were included in the model (1 = Yes, 0 = No) to account for algorithm characteristics. The variables included at level 3 were article characteristics: reference standard (1 = Chart review/clinical data, 0 = Self-report), clinical population (1 = Yes (e.g., HIV patients), 0 = No), study population age (1 = Restricted to only a specific age-group (e.g., 15–45 years [27]), 0 = all ages), and country of data origin (1 = US,0 = non-US). Data structure was not included in the model since it has a strong association with data source; administrative data are generally structured while EMRs can be either structured or unstructured. Test of residual heterogeneity was conducted to find if the heterogeneity not explained by the models is significant or not [28]. To assess model fit, reduction in variance estimates for the models with predictors relative to the initial random-effects pooling models [28] were calculated. An R package metafor [29] was used to conduct the meta-analysis.

### Risk of bias assessment

The articles included in the meta-analysis underwent a risk of bias (ROB) assessment, utilizing the Quality Assessment of Diagnostic Accuracy Studies-2 (QUADAS-2) tool [30]. This tool comprises four domains that evaluate patient selection, index test, reference standard, and flow of patients through the study and timing of the index test(s). Each domain includes specific signaling questions to aid in determining the ROB. For each article, the ROB was evaluated as high, low, or unclear for each domain. A low ROB was assigned if all signaling questions within a domain were answered affirmatively (i.e., best practices were followed). Conversely, a high or unclear ROB was assigned if any signaling question received a negative or unclear response. To ensure accuracy, two reviewers independently performed the ROB assessment on a 5% random training sample of eligible articles. Discrepancies between the reviewers were addressed by a third reviewer, to reach consensus. The remaining articles were then evenly distributed between the two reviewers for ROB assessment.

A sensitivity analysis of the meta-regression models was conducted to assess robustness of the synthesized results. The sensitivity analysis excluded the articles with the presence of high/unclear ROB in any of the four domains of QUADAS-2. A publication bias test was conducted by regressing the null model residuals on their corresponding variance estimates [31].

## Results

### Search results

**Fig. 2:**
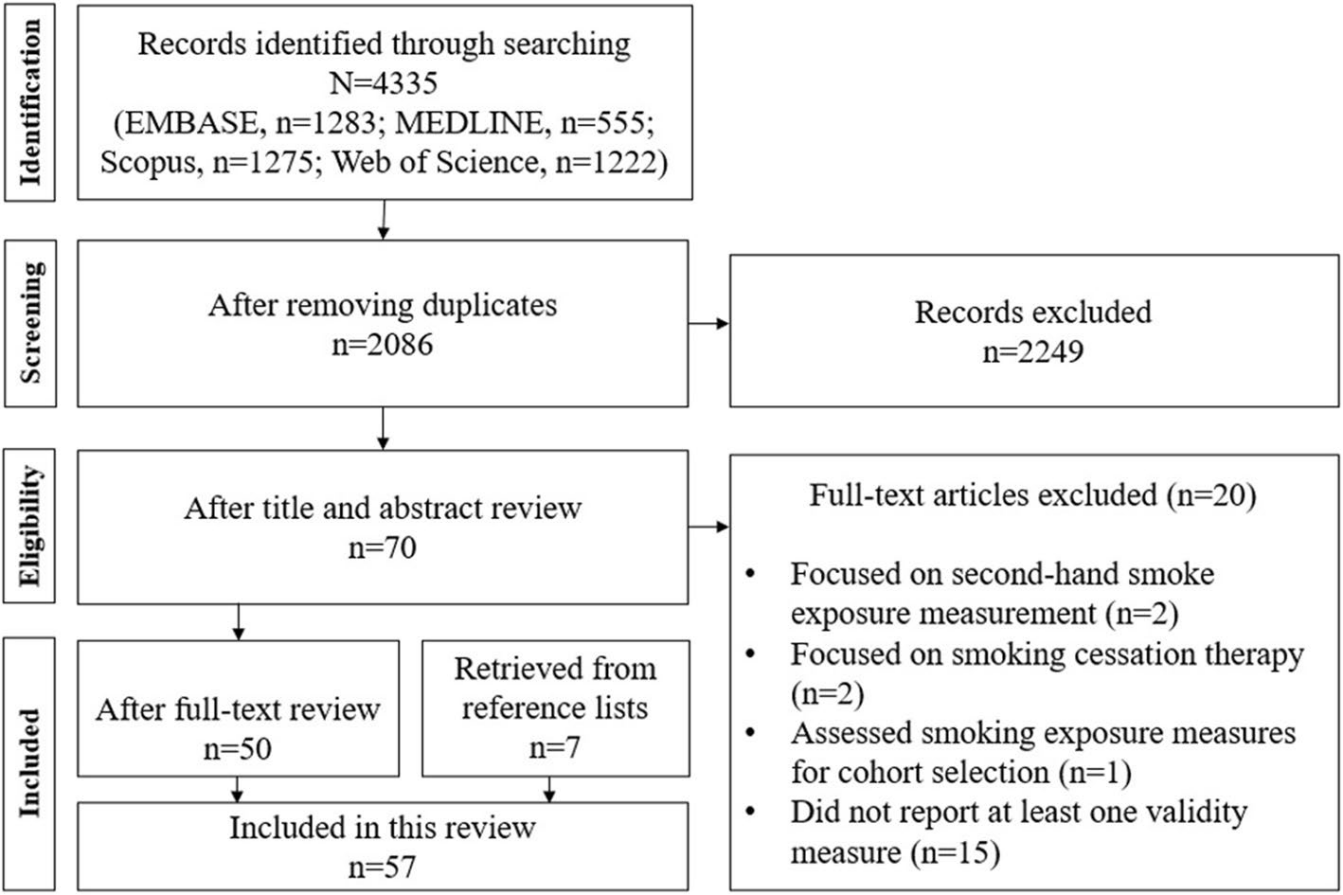
Flowchart of the study selection process

As shown in Fig. 2, a total of 4335 articles were retrieved from the literature search. After removing duplicates, the titles and abstracts of 2086 studies were screened for study inclusion. The screening process left 70 articles for full-text review. Cohen’s kappa for study inclusion/exclusion was 0.97 (95% [CI]: 0.93, 1.00). After full-text review, 20 articles were removed. An additional seven articles were included after the review of reference lists of the remaining 50 articles. Therefore, a total of 57 articles were included in our systematic review (see Additional file 2).

### Article characteristics

Only one (1.8%) of the included articles was published before 2000. The majority of the articles (32, 61.4%) were published after 2014 (Table 1). In most of the articles (44, 77.2%), algorithms were constructed using US data. A large number (37, 64.9%) of articles used clinical population (e.g., lung cancer patients) data. Very few articles (8, 14.0%) reported the use of data from more than one jurisdiction. Overall, the largest number of articles (24, 42.1%) were published in medical informatics/electronic data journals. The majority of the studies (27, 47.4%) validated algorithms using self-report data, followed by (26, 45.6%) chart review data, and (4, 7.0%) clinical data (e.g., serum cotinine in the blood). Only three (5.3%) articles reported that the study population included exclusively a single biological sex group.

**Table 1:** Summary of article characteristics (*n* = 57)

Characteristic	%	Article Reference
**Year of publication**		
≥ 2015	61.4	[9, 12, 27, 32–63]
< 2015	38.6	[10, 64–84]
**Geographical location of data source**		
US	77.2	[9, 10, 27, 33–40, 42–48, 50–55, 57, 59–61, 66–73, 75–83]
Australia	5.3	[64, 65, 74]
UK	3.5	[49, 81]
Canada	3.5	[41, 56]
Other	10.5	[12, 32, 46, 58, 62, 84]
**Clinical population**		
Yes	64.9	[27, 33, 35, 36, 38, 39, 41–48, 52, 53, 55–58, 60–63, 66–68, 70, 72–74, 76–78, 82, 83]
No	35.1	[10, 12, 32, 34, 37, 40, 49–51, 54, 59, 64, 65, 69, 71, 75, 79–81, 84]
**Data from multiple jurisdictions**		
Yes	14.0	[43, 44, 48, 50, 68, 70, 72, 73]
No	86.0	[9, 10, 12, 27, 32–42, 45–47, 49, 51–67, 71, 74–84]
**Journal discipline**		
Medical informatics/Electronic data	42.1	[27, 34, 41, 45, 47–55, 57, 60, 66, 67, 73, 74, 76, 78–80, 83]
Medicine/Clinical	29.8	[35, 36, 38–40, 43, 44, 46, 56, 58, 59, 64, 65, 70, 71, 75, 77]
Public health and epidemiology	10.5	[9, 12, 32, 37, 42, 81]
Health services	8.8	[62, 63, 68, 72, 82]
Substance-related disorders	5.3	[10, 61, 69]
Biomedical	3.5	[33, 84]

### Characteristics of the identified algorithms

The 57 articles reported on validity estimates for 116 algorithms. Overall, 50 (43.1%) algorithms used ICD codes; of this number only 10 used the 10th revision (e.g., tobacco dependence syndrome, personal history of tobacco use disorder) of this classification system and the remainder used the 9th revision (e.g., tobacco use complicating pregnancy, toxic effect of tobacco). Only 11 (9.5%) algorithms used procedure or intervention codes such as advisement to quit and screening for tobacco use followed by an intervention (i.e., smoking cessation program).

Almost three-quarters of the algorithms (83, 71.6%) were constructed using EMR data; the remaining 33 (28.4%) algorithms were constructed using administrative data. Nearly half of the algorithms (54, 46.5%) used structured data such as diagnosis codes, while unstructured EMR data were used to construct 41 (35.3%) algorithms. Only 21 (18.1%) algorithms were based on both structured and unstructured data.

More than half of the algorithms (61, 52.6%) were developed using rule-based methods, such as the presence of any tobacco-related ICD code or a procedure/intervention code in any data source, or the presence of any smoking-related information in inpatient records and/or outpatient medical claims within a defined period of time. The model-based algorithms (*n* = 55) were almost exclusively (53, 96.4%) developed using EMR data. Largest number of the model-based algorithms (24, 43.6%) were developed using natural language processing methods. Specifically, these algorithms were developed by extracting smoking-related information from EMRs and constructing features relevant to smoking status (e.g., former smoker, current smoker), frequency of smoking (e.g., number of cigarettes per day), and temporal information relevant to date or duration (e.g., smoked for 10 years). A total of 16 (29.1%) model-based algorithms were developed using support vector machine models. The remainder (15, 27.3%) used statistical or machine-learning models, such as logistic regression, naïve Bayes, Bayesian networks, neural networks, deep learning methods, and decision trees.

### Validity measures

**Fig. 3:**
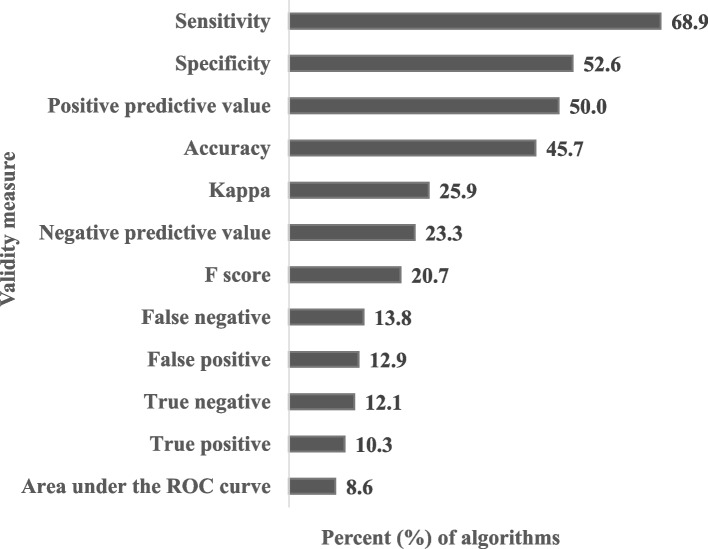
Percent (%) of smoking status algorithms characterized by validity measures (*n* = 116)

Algorithm validity measures reported are depicted in Fig. 3. The number of validation measures reported per algorithm had a median value of 3.0 (IQR = 2). The STARD recommendation of four measures was met for slightly less than half (45.7%) of the identified algorithms. The three most common validity measures were sensitivity (80, 68.9%), specificity (61, 52.6%), and PPV (58, 50.0%). Area under the receiver operating characteristic (ROC) curve (10, 8.6%), true positives (12, 10.3%), and true negatives (14, 12.1%) were the least reported validity measures. The median (IQR) for PPV, sensitivity, and specificity were 88.3% (14.5%), 77.5% (36.5%), and 97.0% (12.0%) respectively.

**Fig. 4:**
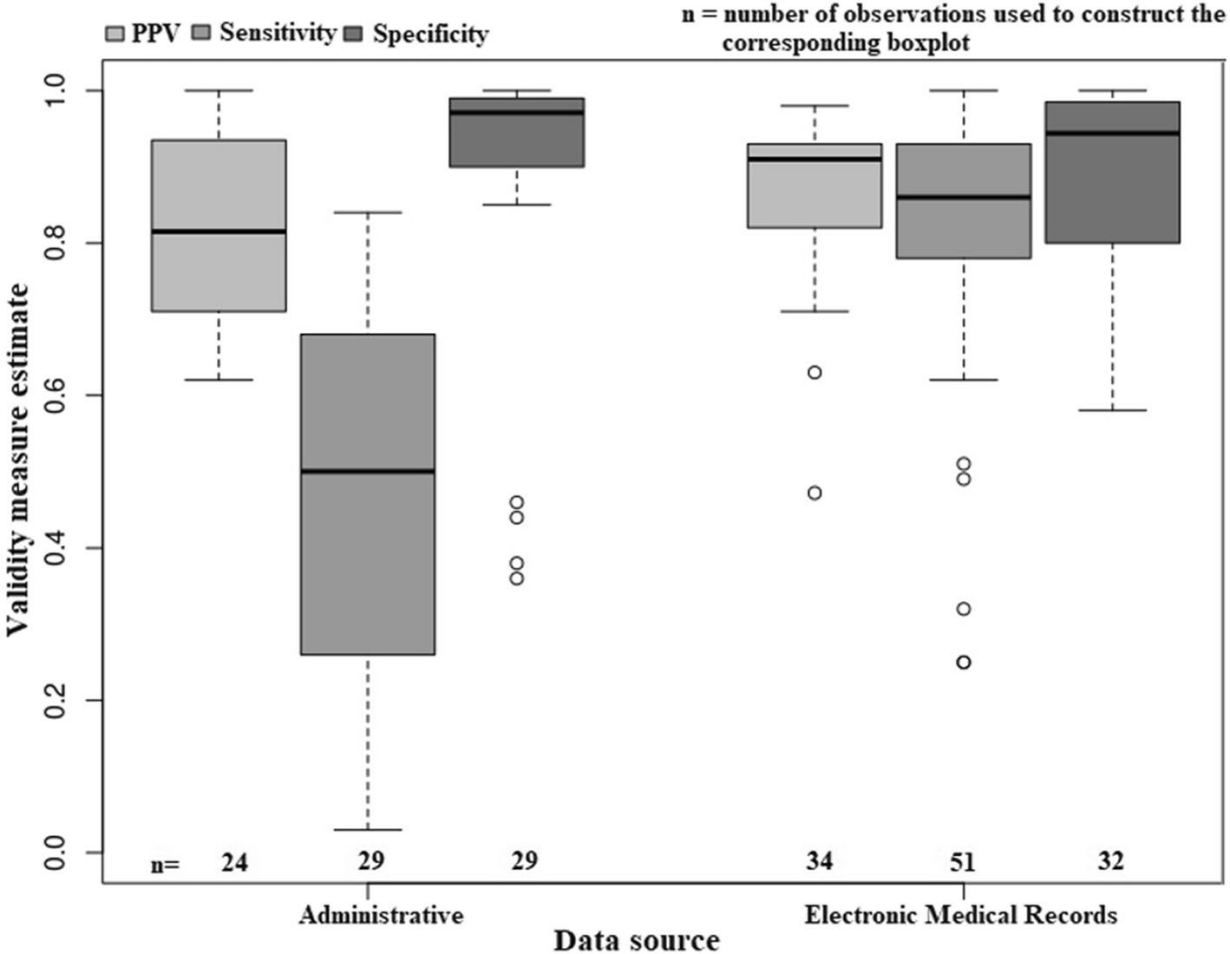
Distribution of selected algorithm validity measures, stratified by algorithm data source. Note: The centre horizontal line within the box represents the median (50th percentile); upper and lower bounds of the box indicate the 25th and 75th percentiles; dashed lines connect the maximum and minimum values; circles represent outliers. PPV = positive predictive value

Figure 4 indicates a negatively skewed distribution of specificity estimates for algorithms constructed using administrative data or EMRs, but not for sensitivity and PPV. The median PPV (91.0%) and sensitivity (86.0%) of the algorithms based on EMR data was higher than for administrative data (PPV = 81.5%, sensitivity = 50.0%). However, median specificity (94.4%) estimate was lower for EMR data than for administrative data (97.1%). For EMR data, variability in estimates of PPV (IQR = 10.5%) and sensitivity (IQR = 15.0%) were lower than for administrative data (IQR for PPV = 23.0% and sensitivity = 42.0%). However, variation in estimates of specificity for EMR data was about twice that of administrative data (IQR 15.2 and 9.0%, respectively).

**Fig. 5:**
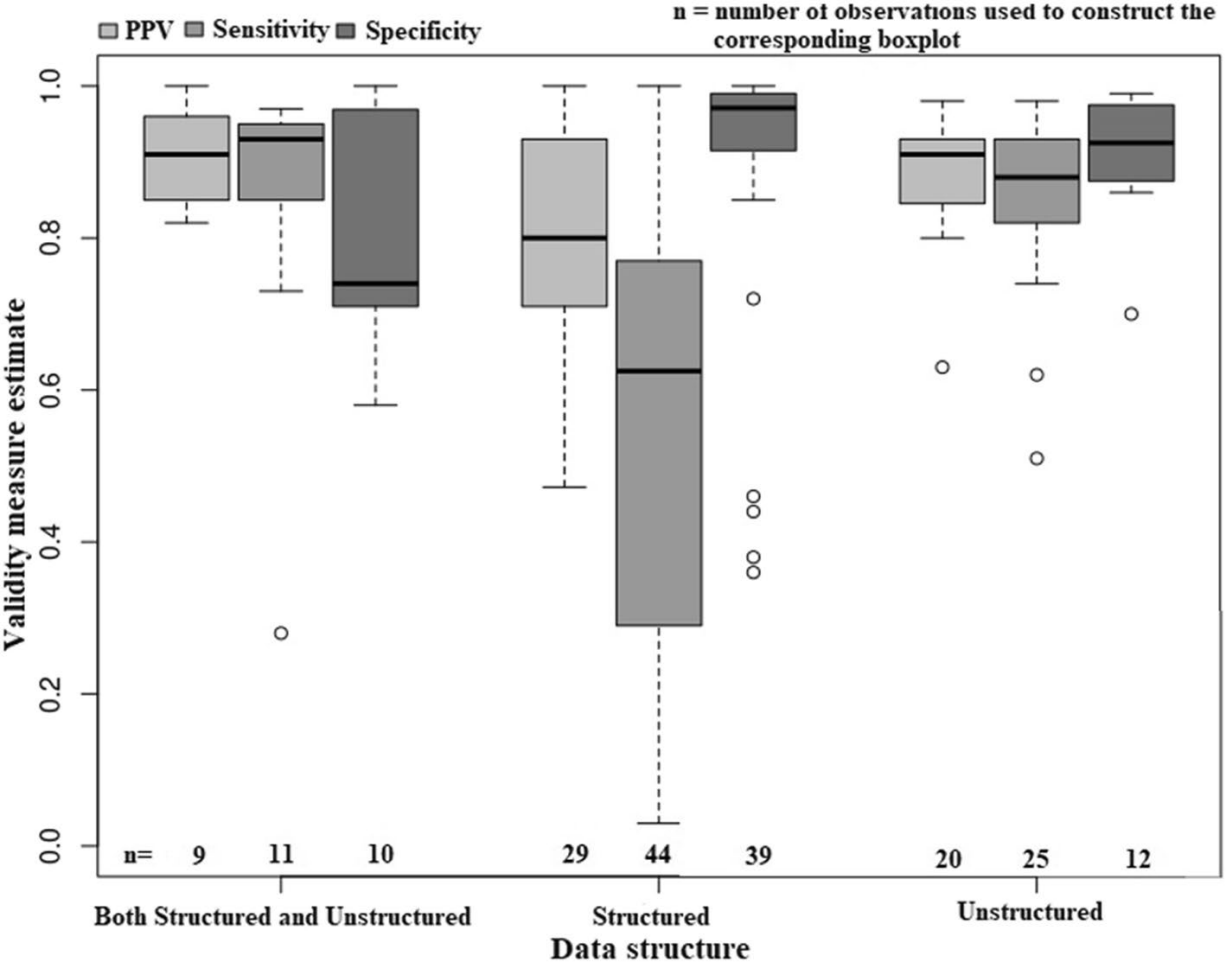
Distribution of selected algorithm validity measures, stratified by algorithm data structure. Note: The centre horizontal line within the box represents the median (50th percentile); upper and lower bounds of the box indicate the 25th and 75th percentiles; dashed lines connect the maximum and minimum; circles represent outliers. PPV = positive predictive value

Figure 5 shows that the distribution of sensitivity was negatively skewed irrespective of data structure, while this was not the case for PPV and specificity. Median PPV and sensitivity estimates for algorithms based on unstructured data (PPV = 91.0%, sensitivity = 88.0%, specificity = 92.5%) were higher than the estimates for algorithms based on structured data (PPV = 80.0%, sensitivity = 62.5%, specificity = 97.1%). Algorithms developed using both structured and unstructured data had PPV and sensitivity estimates similar to algorithms based on unstructured data alone (PPV = 91.0%, sensitivity = 93.0%, specificity = 74.0%). The estimated validity of algorithms based on unstructured data showed less variation (IQR for PPV = 8.2%, sensitivity = 11.0%, and specificity = 9.5%) than algorithms based on structured data (IQR for PPV = 22.0%, sensitivity = 46.5%, and specificity = 7.5%) or that used a combination of structured and unstructured data (IQR for PPV = 11.0%, sensitivity = 10.0%, and specificity = 23.9%).

**Fig. 6:**
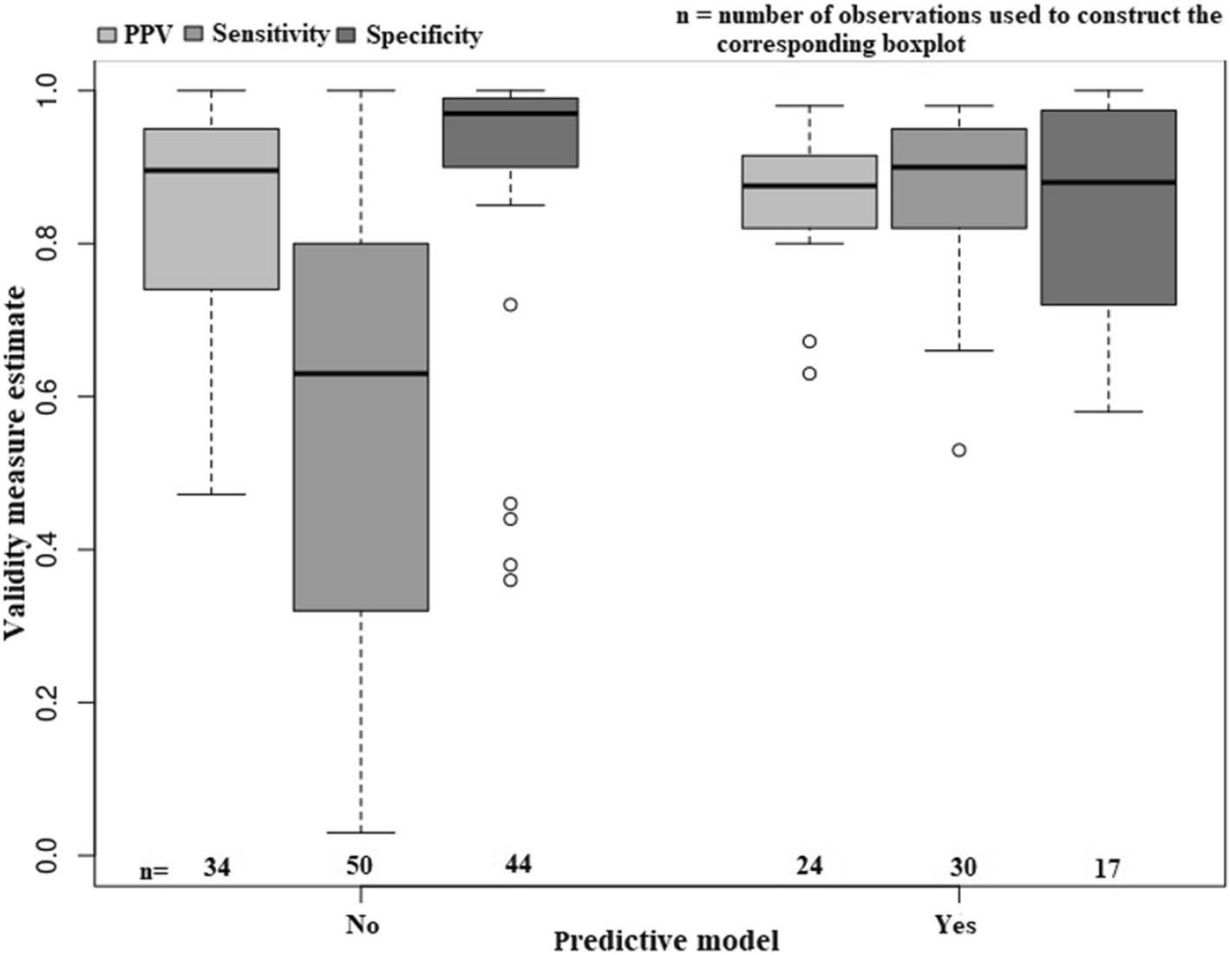
Distribution of selected algorithm validity measures, stratified by use of predictive model in algorithm construction. Note: The centre horizontal line within the box represents the median (50th percentile); upper and lower bounds of the box indicate the 25th and 75th percentiles; dashed lines connect the maximum and minimum with the box; circles represent outliers. PPV = positive predictive value

Regardless of the approach (model-based or rule-based) used to create an algorithm, the distributions of sensitivity and specificity estimates were highly skewed; most of the observations were below their respective average estimates (Fig. 6). Mean sensitivity was 87.3% for model-based algorithms and 57.1% for rule-based algorithms, while mean specificity was 83.9% for model-based algorithms and 90.5% for rule-based algorithms. The median sensitivity for model-based algorithms and rule-based algorithms were 90.0 and 63.0%, respectively. The median PPV and specificity of the algorithms based on predictive models were 87.5 and 88.0% respectively, while algorithms based on deterministic methods had median PPV and specificity of 89.5 and 97.0%, respectively. Variation in PPV (IQR = 9.2%) and sensitivity (IQR = 12.8%) estimates of model-based algorithms was lower than for rule-based algorithms (PPV IQR = 20.5%, sensitivity IQR = 47.5%). Variation in specificity estimates (IQR = 25.4%) was higher for model-based algorithms than rule-based algorithms (IQR = 9.0%).

Estimates of PPV (47 from 20 articles), sensitivity (69 from 34 articles), and specificity (57 from 28 articles) with necessary information to calculate standard error (e.g., CI) were included in the meta-analysis. Three-level model provided significantly better fits compared to two-level models for all three measures PPV (LR = 7.75; *p* = 0.005), sensitivity (LR = 10.180; *p* = 0.001), and specificity (LR = 7.63; *p* = 0.006). The pooled estimates from the null three-level meta-regression models were 84.0% (95% [CI]: 79.0–90.0), 67.0% (95% [CI]: 59.0–76.0), and 92.0% (95% [CI]:87.0–97.0) for PPV, sensitivity, and specificity, respectively. The estimated variances of PPV, sensitivity, and specificity were 0.02, 0.07, and 0.03, respectively. The largest portion of estimated variance was attributable to level 2 in case of sensitivity (64.1%) and specificity (79.7%) followed by level 3 (sensitivity = 35.8%, specificity = 20.3%). However, level 3 (54.8%) accounted for the largest share of estimated variance in PPV followed by level 2 (45.2%). The residual regressions tests (deviation of the intercept from zero) did not indicate the presence of publication bias in the reported estimates of PPV (*p* = 0.753), sensitivity (*p* = 0.471), and specificity (0.124).

Algorithms for EMRs had significantly higher (*p* = 0.001) sensitivity estimates than algorithms for administrative data (Table 2). The model-based algorithms produced significantly higher sensitivity estimates than their rule-based counterparts (*p* = 0.006). The articles including only individuals with certain clinical conditions (e.g., HIV patients) had lower estimates of specificity than articles that used data for general populations (*p* = 0.020). The articles that used data from the US reported significantly larger specificity estimates than the articles based on non-US data (*p* = 0.002). None of the algorithm characteristics and article characteristics included in the model were significantly associated with PPV estimates.

**Table 2:** Meta-regression model parameter estimates (SE) for validity measures of PPV, sensitivity and specificity

Variable	Validity measure					
	PPV	Sensitivity			Specificity	
	Parameter estimate (SE)	*P*-value	Parameter estimate (SE)	*P*-value	Parameter estimate (SE)	*P*-value
**Data source**						
EMR	−0.076 (0.073)	0.304	**0.251** **(0.076)**	0.001	−0.057 (0.054)	0.297
Administrative	Ref		Ref		Ref	
**Predictive model**						
Yes	−0.017 (0.076)	0.826	**0.195** **(0.069)**	0.006	−0.094 (0.052)	0.078
No	Ref		Ref		Ref	
**Reference standard**						
Chart review/clinical data	0.095 (0.072)	0.198	0.086 (0.082)	0.297	−0.025 (0.059)	0.672
Self-report	Ref		Ref		Ref	
**Clinical population**						
Yes	0.108 (0.063)	0.144	−0.096 (0.081)	0.239	**− 0.140 (0.058)**	0.020
No	Ref		Ref		Ref	
**Study population age**						
Restricted	0.133(0.067)	0.054	−0.016(0.062)	0.793	0.035(0.045)	0.441
All ages	Ref		Ref		Ref	
**Country of data origin**						
US	−0.006(0.072)	0.937	− 0.083(0.079)	0.304	**0.195(0.059)**	0.002
non-US	Ref		Ref		Ref	

Boldface font denotes a statistically significant estimate; *EMR* electronic medical record, *SE* standard error, *PPV *positive predictive value

The tests of residual heterogeneity in the models for all three measures suggest statistically significant amounts of unexplained heterogeneity in their estimates (*p* < 0.0001). The models with predictors had lower variances compared to the initial random-effects pooling model by 41.1 and 16.0%, respectively, for sensitivity and specificity. However, the estimated PPV variance remained unchanged in the null model and the model with the predictors. The predictors included in our models were able to explain the variability in sensitivity and specificity better than the respective null models. However, the predictors did not add any value when explaining variation in the PPV estimates.

The results of the ROB assessment are summarized in Table 3. In total, 38 articles from the meta-regression models were evaluated for ROB using the QUADAS-2 tool. The majority of these articles (29, 76.3%) were deemed to have a low ROB across all four domains. High ROB was observed in three articles, with one in the index test domain and two in the flow and timing domain. Additionally, ROB was unclear in four articles in the reference standard domain and three articles in the patient selection domain.

**Table 3:**
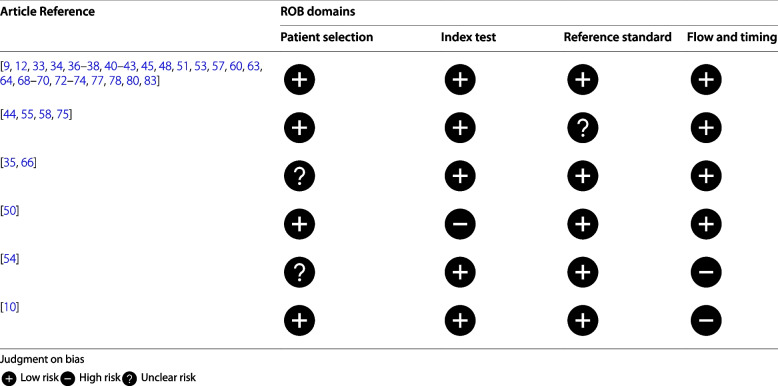
Risk of bias (ROB) assessment results [9, 10, 12, 33–38, 40–45, 48, 50, 51, 53–55, 57, 58, 60, 63, 64, 66, 68–70, 72–75, 77, 78, 80, 83]

The PPV, sensitivity, and specificity meta-regression models, respectively, had two articles (containing 4 algorithms), nine articles (containing 14 algorithms), and five articles (containing 5 algorithms) with high/unclear ROB. A sensitivity analysis that removed these articles with potential bias produced results consistent with the primary meta-analysis (Additional file 3).

## Discussion

Smoking status is an important covariate in many disease risk prediction models and trends in smoking status are of interest in epidemiologic studies [85–87]. Electronic health databases can be leveraged to develop prediction models [88] and surveillance estimates that include smoking status information. Validation studies are important to assess the quality of electronic health data to ascertain a variety of individual characteristics, including smoking status [89]. Validation studies of electronic health data sources have been synthesized for chronic diseases such as diabetes [90], cancer [91], and social determinants of health (e.g., ethnicity, occupation) [92], but the validity of smoking status algorithms constructed from electronic health databases is a gap in the literature.

We found that a large number of validation studies for smoking status algorithms used electronic health data from the US; a similar trend was reported in systematic reviews for other validation studies, such as for comorbidity indices [93] and kidney disease [94]. Canadian provinces and territories collect comprehensive administrative health data [95]. Additionally, many provinces collect EMR data through the Canadian Primary Care Sentinel Surveillance Network [96] or other activities [97]. However, this study identified only two articles that used Canadian data. Similarly, we found very few articles used data from European countries, including the Scandinavian countries, which also have comprehensive administrative and EMR data [98, 99]. The vast majority of the algorithms were constructed with data from a single jurisdiction, potentially limiting the transportability of these algorithms across jurisdictions. At the same time, this finding is not unexpected, given that it can be challenging to find a comparable validation data source in more than one jurisdiction. A large number of studies used medical chart review to validate the algorithms, a result comparable to a systematic review of algorithms for obesity [100].

The median number of validity measures reported per algorithm was below the STARD recommendation of at least four measures. Overall, the sensitivity estimates were lower than the estimates of PPV and specificity. This finding is in line with a study that reviewed validation studies of algorithms to identify obesity in administrative databases [100]. Similar to other reviews focusing on chronic conditions [101], infections [102], and nonmedical opioid use [103]; considerable variation was found in the estimates for the reported algorithm validity measures such as PPV, sensitivity, and specificity overall and by selected algorithm characteristics. The model-based algorithms tended to have less variable PPV and sensitivity estimates than the rule-based algorithms. This suggests the model-based algorithms had more consistent performance in terms of accurately predicting positive cases (i.e., PPV) and capturing true positives (i.e., sensitivity). However, the model-based algorithms were more variable in specificity estimates compared to the rule-based algorithms. This may suggest the complexity of model-based algorithms affected specificity in different study-specific scenarios. Model-based smoking status ascertainment algorithms had better performance than rule-based algorithms, which is in contrast to findings from a rheumatoid arthritis validation study that utilised these two approaches and found similar predictive performance [104]. Nevertheless, another study demonstrated that model-based algorithms can improve sensitivity and specificity estimates over the estimates from rule-based algorithms for classifying carotid endarterectomy indication using physician claims and hospital discharge data [105]. Residual confounding due to data source may remain in the summarized relationship between validity measures and the use of a predictive model. This is particularly noteworthy, because a majority of model-based algorithms were constructed using EMR data. These data capture more detailed and comprehensive clinical information compared to administrative data, resulting in better performance than the rule-based algorithms that relied on administrative data. The model for sensitivity suggested significant difference in algorithms for EMRs and administrative data, in contrast to findings from an earlier study to predict binary outcomes such as 1 year mortality and hospital readmission [106]. The specificity model indicated considerably lower estimates for algorithms belonging to articles focusing only on clinical population and higher estimates for articles using US data. Class imbalance may be partially responsible for these findings on specificities [107]. No significant difference was detected in the results of PPV model. Overall, the fitted models with predictors did not adequately explain heterogeneity in validity measure estimates. This finding may be attributed to factors identified as sources of heterogeneity, such as the use of alternative coding methods and misclassified diagnosis by examining medications prescribed for different purposes, in the studies included in our meta-analysis [108]. Many articles in our meta-analysis had low ROB, but a review of algorithms for neurodevelopmental disorders had contrasting results [109]. The key findings from the meta-analysis did not change after a sensitivity analysis that excluded articles considering the ROB. A similar result was observed in a meta-analysis of articles using machine-learning to predict the spread of breast cancer to armpit lymph nodes [110]. Our analysis did not find strong evidence of publication bias in the pooled estimates of validity measures from null models. This finding should be interpreted with caution considering the limitations of linear regression [111]. For example, the relationship between residuals and variances may be non-linear, which can lead to inaccurate assessment of publication bias.

### Strengths and limitations

The strengths of this systematic review and meta-analysis include the breadth of citation databases that we searched, the wide variety of article characteristics, and the detailed analysis of extracted information at the algorithm level. We identified statistically significant sources of variation in the estimates of sensitivity (data source and use of predictive model) and specificity (patient characteristics and country of data origin). However, we recognize that this study is not without limitations. Articles published in languages other than English and grey literature, such as government reports and graduate dissertations, were excluded. These exclusions may affect the generalizability of our findings. To mitigate this gap, the reference lists of the included articles were searched for additional articles. A large portion of the variation in the reported estimates of PPV, sensitivity, and specificity remained unexplained in the meta-regression models. To evaluate the reliability of the results of these models, a sensitivity analysis was performed incorporating the findings from the ROB assessment.

## Conclusions

Evidence syntheses of algorithm validation studies have often focused on chronic or infectious disease case ascertainment and social determinants of health [90–92]. This study contributes to the body of literature about validation studies and examines a relatively unexplored area of behavioral risk factor algorithms for electronic health databases.

We found that numerous algorithms have been developed to identify smoking status in electronic health databases. The identified algorithms vary in terms of data source, data structure, and methods of construction. In general, the algorithms had high specificity and low sensitivity when predicting smoking status, although there is evidence that sensitivity can be improved by using EMR data and predictive models to construct the algorithms.

A number of opportunities exist to develop algorithms to measure smoking status using population-based electronic health data. For example, combining multiple data sources, including EMR and administrative data, may produce algorithms with high sensitivity [112]. The breadth of the longitudinal information [22] available in the electronic health databases can be utilized to develop algorithms. Methods such as longitudinal discriminant analysis [113] and semiparametric mixed-effects model [114] can be used to construct algorithms based on longitudinal data. The application of ensemble machine learning classification models and use of large language models (LLMs) remained unexplored in this line of research. Ensemble machine learning involves combining individual models to improve overall predictive performance. For example, random forest ensemble classifiers [115, 116] may be used to identify smoking status in electronic health databases. These classifiers may have reduced potential over-fitting of the model and improve performance measures relative to decision trees [117]. LLMs are trained deep-learning models that understands and generates text in a human-like fashion [118]. These models can be deployed to identify smoking status from text-based unstructured EMR data.

### Supplementary Information

**Additional file 1.** Search strategy.

**Additional file 2.** Article Characteristics.

**Additional file 3. **Sensitivity analysis.

**Additional file 4.** Preferred Reporting Items for Systematic Reviews and Meta-Analyses (PRISMA) checklist.

## Data Availability

The datasets used and/or analyzed during the current study are available from the corresponding author on reasonable request.

## Abbreviations

EMR: Electronic medical record
PRISMA: Preferred Reporting Items for Systematic Reviews and Meta-Analyses
CI: Confidence interval
ICD: International Statistical Classification of Diseases and Related Health Problems
STARD: Standards for Reporting Diagnostic Accuracy
IQR: Interquartile range
ROB: Risk of bias
PPV: Positive predictive value
ROC: Receiver operating characteristic
