# Supplementary material for: The validity of electronic health data for measuring smoking status: a systematic review and meta-analysis
Source: BMC Med Inform Decis Mak. 2024 Feb 2;24:33. doi: 10.1186/s12911-024-02416-3 (PMC10836023; doi:10.1186/s12911-024-02416-3)
Supplement: Supplementary file 1 — Additional file 1. Search strategy. [file 12911_2024_2416_MOESM1_ESM.docx]

**Additional file 1:** Search strategy

Ovid interface: EMBASE

Search Date: 11/22/2022

| **Search Line** | **Search Terms** | **Results** |
| --- | --- | --- |
| **1** | (valid* or quality or accuracy or sensitivity or specificity).ti,ab,kf. | 4721416 |
| **2** | ("electronic medical record*" or "electronic health record*" or "administrative health data" or "administrative data" or "health care data" or "administrative billing record*" or "administrative claims data" or "claims data" or "hospital data" or "hospital discharge data" or "medicare data" or "medicaid data").ti,ab,kf. | 142981 |
| **3** | (smok* or Tobacco).ti,ab,kf. | 556655 |
| 4 | 1 AND 2 AND 3 | 1330 |
| 5 | Limit 4 to (English language and yr=”1990-Current”) | 1283 |

Ovid Interface: MEDLINE

Search Date: 11/22/2022

| **Search Line** | **Search Terms** | **Results** |
| --- | --- | --- |
| **1** | (valid* or quality or accuracy or sensitivity or specificity).ti,ab,kf. | 3554243 |
| **2** | ("electronic medical record*" or "electronic health record*" or "administrative health data" or "administrative data" or "health care data" or "administrative billing record*" or "administrative claims data" or "claims data" or "hospital data" or "hospital discharge data" or "medicare data" or "medicaid data").ti,ab,kf. | 83536 |
| **3** | (smok* or Tobacco).ti,ab,kf. | 386090 |
| 4 | 1 AND 2 AND 3 | 581 |
| 5 | Limit 4 to (English language and yr=”1990-Current”) | 555 |

Scopus

Search Date: 11/22/2022

| **Search Line** | **Search Terms** | **Results** |
| --- | --- | --- |
| **1** | TITLE-ABS-KEY(valid* or quality or accuracy or sensitivity or specificity) | 8299452 |
| **2** | TITLE-ABS-KEY("electronic medical record*" or "electronic health record*" or "administrative health data" or "administrative data" or "health care data" or "administrative billing record*" or "administrative claims data" or "claims data" or "hospital data" or "hospital discharge data" or "medicare data" or "medicaid data") | 109444 |
| **3** | TITLE-ABS-KEY(smok* or Tobacco) | 539714 |
| 4 | 1 AND 2 AND 3 | 1394 |
| 5 | Limit 4 to (Language=English AND Pubyear=1990 to Present) | 1275 |

Web of Science

Search Date: 11/22/2022

| **Search Line** | **Search Terms** | **Results** |
| --- | --- | --- |
| **1** | TS=(valid* or quality or accuracy or sensitivity or specificity) | 8087727 |
| **2** | TS=("electronic medical record*" or "electronic health record*" or "administrative health data" or "administrative data" or "health care data" or "administrative billing record*" or "administrative claims data" or "claims data" or "hospital data" or "hospital discharge data" or "medicare data" or "medicaid data") | 102195 |
| **3** | TS=(smok* or Tobacco) | 620314 |
| 4 | 1 AND 2 AND 3 | 1263 |
| 5 | Refine 4 by (Languages=English and publication Years=1990-Current) | 1222 |
