# Supplementary material for: The validity of electronic health data for measuring smoking status: a systematic review and meta-analysis
Source: BMC Med Inform Decis Mak. 2024 Feb 2;24:33. doi: 10.1186/s12911-024-02416-3 (PMC10836023; doi:10.1186/s12911-024-02416-3)
Supplement: Supplementary file 3 — Additional file 3. Sensitivity analysis. [file 12911_2024_2416_MOESM3_ESM.docx]

**Additional file 3:** Sensitivity analysis

**Table A1** Meta-regression model parameter estimates (SE) for validity measures of PPV, sensitivity, and specificity

| **Variable** | Validity measure | | | | | | |
| --- | --- | --- | --- | --- | --- | --- | --- |
|  | PPV | | Sensitivity | | | Specificity | |
|  | Parameter estimate (SE) | P-value | | Parameter estimate (SE) | P-value | Parameter estimate (SE) | P-value |
| **Data source** |  |  | |  |  |  |  |
| EMR | -0.096(0.095) | 0.319 | | **0.257(0.092)** | 0.007 | -0.063(0.059) | 0.299 |
| Administrative | Ref |  | | Ref |  | Ref |  |
| **Predictive model** |  |  | |  |  |  |  |
| Yes | -0.003(0.112) | 0.979 | | **0.187(0.086)** | 0.034 | -0.118(0.062) | 0.064 |
| No | Ref |  | | Ref |  | Ref |  |
| **Reference standard** |  |  | |  |  |  |  |
| Chart review/clinical data | 0.088(0.077) | 0.261 | | 0.088(0.092) | 0.342 | -0.039(0.063) | 0.540 |
| Self-report | Ref |  | | Ref |  | Ref |  |
| **Clinical population** |  |  | |  |  |  |  |
| Yes | 0.175(0.097) | 0.079 | | -0.045(0.099) | 0.648 | **-0.152(0.065)** | 0.025 |
| No | Ref |  | | Ref |  | Ref |  |
| **Study population age** |  |  | |  |  |  |  |
| Restricted | -0.112(0.072) | 0.131 | | -0.018(0.077) | 0.814 | 0.050(0.052) | 0.344 |
| All ages | Ref |  | | Ref |  | Ref |  |
| **Country of data origin** |  |  | |  |  |  |  |
| US | -0.037(0.095) | 0.702 | | -0.066(0.100) | 0.511 | **0.208(0.063)** | 0.002 |
| non-US | Ref |  | | Ref |  | Ref |  |
| Note: boldface font denotes a statistically significant estimate; EMR = electronic medical record; SE=standard error; PPV= positive predictive value | | | | | | | |

**Results from the null model**

The null three-level meta-regression models produced pooled estimates for PPV, sensitivity, and specificity of 0.849 (95% [CI]: 0.788-0.911), 0.658 (95% [CI]: 0.557-0.758), and 0.915 (95% [CI]: 0.857-0.973) respectively. The estimated variances for these measures were 0.018 (PPV), 0.075 (sensitivity), and 0.027 (specificity). Level 2 accounted for most of the estimated variance in sensitivity (61.2%) and specificity (79.2%), with level 3 contributing to a lesser extent (sensitivity=38.7%, specificity=20.8%). In contrast, level 3 accounted for the largest share of estimated variance in PPV (62.4%), followed by level 2 (37.6%). The residual regressions tests (deviation of the intercept from zero) suggest the absence of publication bias in the reported estimates of PPV (p=0.646), sensitivity (p=0.552), and specificity (0.117). The introduction of predictors in the models led to a reduction in heterogeneity variance for PPV, sensitivity, and specificity by 5.3%, 37.3%, and 18.5%, respectively, compared to the null model.
